# Concomitant necrotizing encephalitis and granulomatous meningoencephalitis in four toy breed dogs

**DOI:** 10.3389/fvets.2022.957285

**Published:** 2022-09-01

**Authors:** Jasmin Nicole Nessler, Anna Oevermann, Marina Schawacht, Ingo Gerhauser, Ingo Spitzbarth, Sophie Bittermann, Frank Steffen, Martin Jürgen Schmidt, Andrea Tipold

**Affiliations:** ^1^Department for Small Animal Medicine and Surgery, University of Veterinary Medicine Hannover, Hannover, Germany; ^2^Department of Clinical Research and Veterinary Public Health, Vetsuisse Faculty, University of Bern, Bern, Switzerland; ^3^Department of Pathology, University of Veterinary Medicine Hannover, Hannover, Germany; ^4^Department of Clinical Neurology, Vetsuisse Faculty, University of Zurich, Zurich, Switzerland; ^5^Clinic for Small Animal-Surgery, Department of Veterinary Clinical Sciences, Faculty of Veterinary Medicine, Justus-Liebig-University, Giessen, Germany

**Keywords:** meningoencephalitis of unknown origin (MUO), necrotizing meningoencephalitis, necrotizing leukoencephalitis, canine (dog), inflammatory brain disease, histopathology (HPE), granulomatous meningoencephalitis (GME)

## Abstract

The term “meningoencephalitis of unknown origin” (MUO) describes a group of different encephalitides in dogs in which no infectious agent can be identified and a multifactorial etiology is suspected. Among others, genetic factors and unknown triggers seem to be involved. Included are necrotizing leukoencephalitis (NLE), necrotizing meningoencephalitis (NME), and granulomatous meningoencephalitis (GME). In this case series, we describe the histopathological findings of four toy breed dogs with focal or multifocal necrotizing encephalitis and mainly lymphocytic perivascular infiltrates on histopathological examination. At the same time, however, in all dogs, focal or multifocal high-grade angiocentric granulomatous inflammatory lesions were evident with focal histiocytic perivascular infiltrates in the brain. The former changes are typical for NLE and NME. In contrast, the latter changes are indicative of GME. This case series shows that the boundaries between the necrotizing and granulomatous variants of MUO might be smooth and suggests that NLE, NME, and GME are not as distinct as previously described. This finding could be a crucial piece of the puzzle in the study of the pathogenesis of MUO as individual susceptibility and specific triggers could be responsible for the manifestation of the different MUO subtypes.

## Introduction

Granulomatous meningoencephalitis (GME), necrotizing meningoencephalitis (NME), and necrotizing leukoencephalitis (NLE) are supposed to be subtypes of canine meningoencephalitis of unknown origin (MUO) (1, 2). MUO seems to be multifactorial: a trigger is suspected to cause exaggerated immune response in susceptible individuals, but both the trigger and the underlying individual predisposition remain mostly elusive (1), though a genetic defect is suspected to predispose to NME in some toy breeds (3). Typically, patients display progressive clinical signs of focal or multifocal central nervous system (CNS) dysfunction, with focal to multifocal CNS lesions in advanced imaging and often non-suppurative cerebrospinal fluid (CSF) pleocytosis, indicating CNS inflammation. An infectious agent can be found neither in the CSF nor in the neuronal parenchyma (4–9), and immunomodulatory therapy often improves clinical signs (2, 10, 11).

Although signalment, history, clinical signs, cross-sectional imaging modalities of the brain and CSF examination, and exclusion of infectious agents may allow a tentative diagnosis of MUO *ante mortem* (9, 10), different subtypes can often not be separated (2). Thus, the gold standard for diagnosing different types of MUO still remains histopathology (1).

Findings in histopathology are reported to be relatively specific for each subtype: GME is characterized by angiocentric or nodular granulomatous lesions due to focal eccentric nodular proliferation of macrophages within histiocytic perivascular cuffs in the Virchow-Robin space (12) in the cerebellum, medulla oblongata, and spinal cord. In contrast, non-suppurative perivascular inflammation and necrotic lesions of NLE occur mostly in the white matter of the cerebrum and brain stem, while in NME, they affect predominantly the gray matter and meninges of the telencephalon (12, 13).

Due to frequent overlap of histopathological and clinical features as well as MRI findings, NME and NLE are often taken together as necrotizing encephalitis (NE), and the authors of several reviews have speculated that NME and NLE are even just different manifestations of the same disease entity with breed specific characteristics (1, 14).

While NLE is mostly found in young Yorkshire Terriers and French Bulldogs (14–16), NME appears predominantly in young Maltese, Chihuahua, and Pug dogs (3, 17). GME, on the contrary, might occur in any breed at any age, although preferably in young to middle-aged toy breed dogs (2).

Till present, it is still under debate whether all subtypes of MUO belong to an array of different manifestations within the same disease entity or if they display completely different diseases (1).

This multicentric case study presents histopathological findings of four toy breed dogs, each with overlapping phenotypes of NME/NLE and GME.

## Materials and methods

All clinical examinations were performed with the informed written owner's consent by a resident or diplomate of the European College of Veterinary Neurologist (ECVN). Anesthesia was induced with levomethadone, diazepam, and propofol and maintained by the administration of isoflurane in oxygen-nitrous oxide or air. The magnetic resonance imaging (MRI) examination of the brain was performed under general anesthesia with the dog in sternal or dorsal recumbency using 0.3 or 3.0 Tesla (Philips, Achieva 3.0T TX MRI, Phillips Healthcare, Hamburg, Germany, or Hitachi Airis II, Hitachi Medical Systems, Düsseldorf, Germany) and consisted of turbo spin echo sequences with T2-weighted (T2W), fluid attenuation inversion recovery T2 (T2W FLAIR), and T1-weighted images (T1W) before and after intravenous administration of MRI contrast (Gadolinium-Dotarem, 0.2 mmol/kg, or Gadopentetate Dimeglumineb, 0.1 mmol/kg). Computed tomography (CT) was performed using Siemens Somatom AR.T (Siemens Switzerland AG, Fahrweid, Switzerland) pre- and postintravenous administration of iodinated contrast medium.

Necropsies and histopathology of formalin-fixed and paraffin-embedded tissues were performed at the Department of Pathology of the University of Veterinary Medicine Hannover, Foundation, and the Department of Clinical Research and Veterinary Public Health, Vetsuisse Faculty, University of Bern. All cases were investigated by a diplomate of the European College of Veterinary Pathologists (ECVP). Brain and spinal cord were fixed in 10% neutral buffered formalin, processed, embedded in paraffin, sectioned at 4 μm, and stained with hematoxylin and eosin (H&E) in all dogs. Immunohistochemistry was performed to exclude an infection with rabies virus, canine distemper virus, *Neospora caninum*, and *Toxoplasma gondii* using the avidin-biotin-complex (ABC)-method with 3,3′-diaminobenzidine (DAB) as chromogen (6, 18–20). Selected sections of the brain were stained with Grocott's methenamine silver, Gram, Periodic acid–Schiff (PAS), or Ziehl-Neelsen (20).

Dogs were included if, on histopathological examination, concomitant signs of NME or NLE and GME were evident, which are multifocal asymmetric necrosis with non-purulent inflammation predominantly in the gray or in the white matter of the cerebrum and thalamus for NME or NLE, respectively, and additional angiocentric lymphoplasmacellular or granulomatous inflammatory infiltration predominantly in the white matter of the brain stem, cerebellum, and spinal cord for GME (12, 13), and no infectious agent could be found. Data banks from 2012 to 2021 of both institutes were searched. In total, 65 dogs with histopathological signs of (meningo-)encephalitis without a known infectious agent were identified (Table 1), of which 4 dogs matched the inclusion criteria of concomitant signs of NME or NLE and GME.

**Table 1:** All cases of dogs with meningoencephalitis of unknown origin (MUO) examined in both pathological institutes from 2010 to 2021.

**Histopathological diagnosis**	**Number of dogs**
GME	22
NLE	9
NME	14
Concomitant findings of NME and NLE	1
Lymphohistiocytic MUO (findings not consistent with GME, NME or NLE)^a^	12
MUO with CNS vasculitis^b^	3
Concomitant findings of GME and NLE/NME	4
**Total**	**65**

GME, granulomatous meningoencephalitis; NLE, necrotizing leukoencephalitis; NME, necrotizing meningoencephalitis; MUO, meningoencephalitis of unknown origin; CNS, central nervous system; ^a^as described in (21); ^b^dogs described in (22).

## Case descriptions

### Case 1

A 6.5-year-old male neutered Yorkshire Terrier was presented with a 3-week history of progressive gait abnormality in the pelvic limbs, two suspected seizures with tremor, restlessness, and increased water intake, according to the owner.

On presentation, the general physical examination was normal with respiratory sinus arrhythmia. In the neurological examination, the dog had mild kyphotic posture, paraparesis accentuated on the left side, and a reduced to absent paw placement of the left pelvic limb. The dog also had a reduced to absent menace response bilaterally and a ventromedial strabismus of the left eye. Based on these findings, a multifocal meningoencephalopathy was suspected.

On complete blood count (CBC), mild leukopenia (5.32 × 103/μl, reference range 6–12 × 103/μl) was evident, the serum biochemistry profile was within reference ranges.

**Figure 1:**
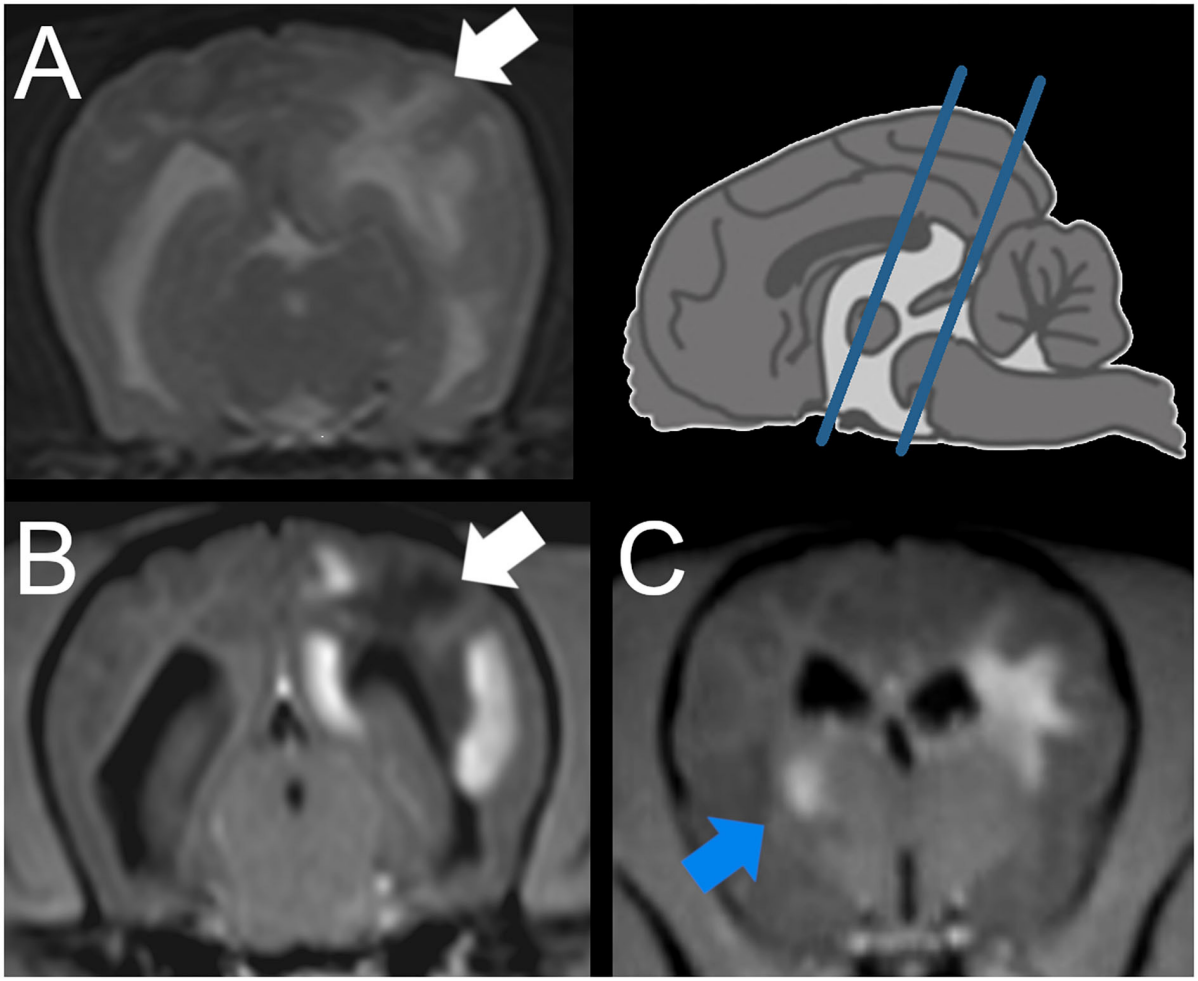
Magnetic resonance imaging (MRI) of a Yorkshire Terrier with meningoencephalitis of unknown origin (case 1). Transverse plane at the level of the mesencephalon **(A,B)** and interthalamic adhesion **(C)** (see blue lines in the schematic overview upper right), A: T2 weighted (w), B, C: T1w post contrast injection. Intraaxial lesion of the subcortical white matter of the cerebral hemisphere, identified on histopathology as predominantly necrotic (**A,B**: white arrow). Intraaxial lesion of the striated body identified as granulomatous lesion (**C**: blue arrow).

On MRI of the brain (Figure 1), multifocal bilateral asymmetric forebrain lesions were seen, mainly affecting the subcortical white matter but also the gray matter of the left parietal lobe, the right occipital lobe, the left hippocampus, and thalamus with no to mild mass effect. The lesions were hyperintense in T2W and FLAIR and hypointense in T1W and were mostly well demarcated. In the lesions, heterogeneous strong contrast enhancement was present.

The suboccipital CSF sample contained mildly increased total protein of 34 mg/dl (reference range <25 mg/dl), lymphocytic pleocytosis (270 cells/μl, reference range <5 cells/μl; 84% lymphocytes, 12% monocytes, and 4% macrophages), and mild suspected iatrogenic blood contamination (190 cells/μl, reference range 0 cells/μl) was evident. MUO was suspected, but the owners denied any further examinations and elected euthanasia.

**Figure 2:**
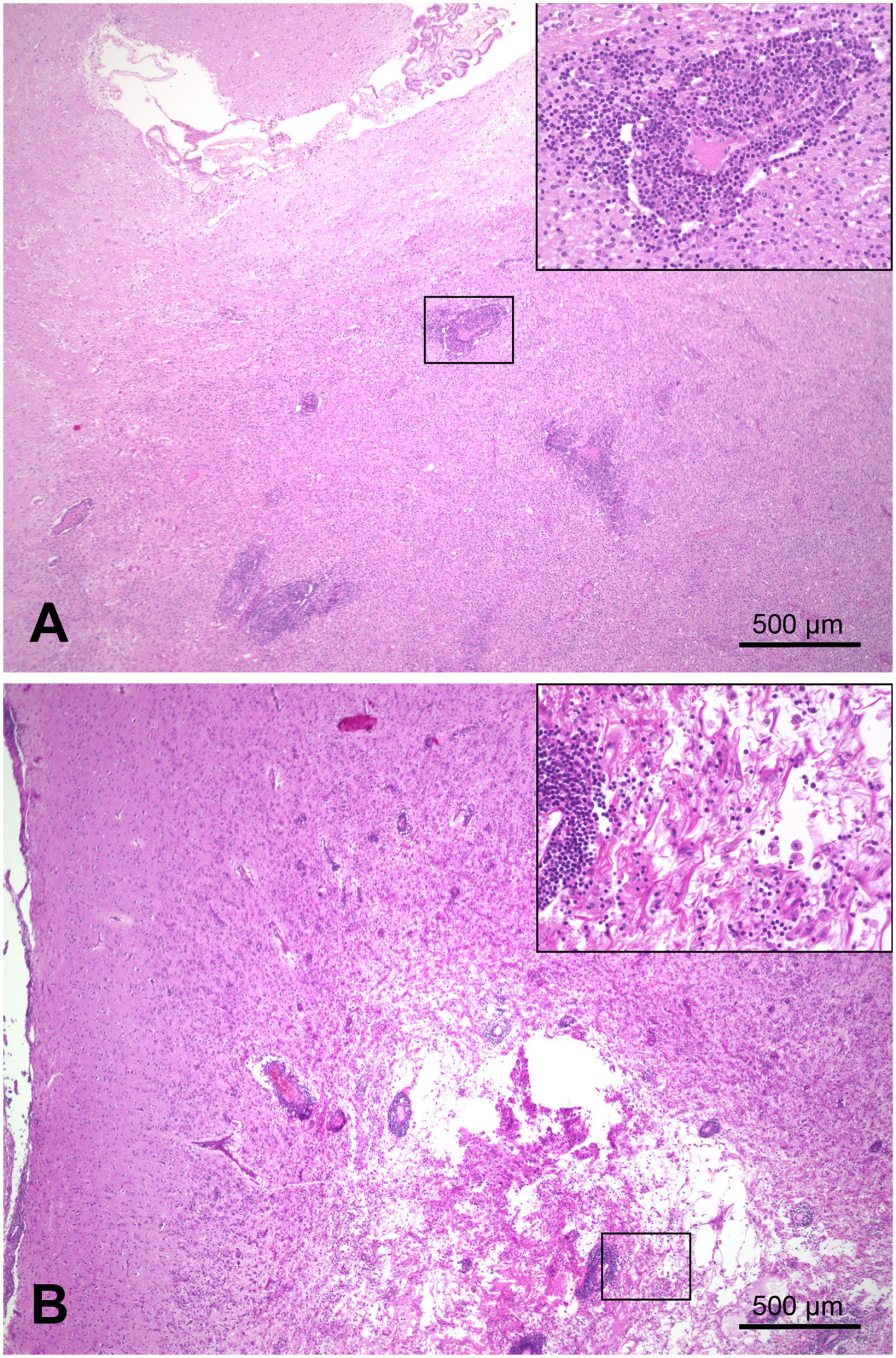
Combined necrotizing leukoencephalitis and granulomatous meningoencephalitis in the brain of a Yorkshire Terrier (case 1). **(A)** right striatal body; marked granulomatous inflammation with severe lymphocytic perivascular cuffing. **(B)** cerebrum, left parietal lobe; marked necrotizing leukoencephalitis in subcortical white matter with gitter cells (malacia) and severe lymphohistiocytic perivascular cuffing; bars = 500 μm.

On pathological examination of the brain, multifocal marked necrotizing leukoencephalitis was evident with many gitter cells (malacia) and severe lymphohistiocytic perivascular cuffing in the subcortical white matter of the left parietal lobe (Figure 2). A moderate lymphohistiocytic inflammation with severe gliosis, gemistocytes, dilated myelin sheaths, and spheroids was found in the periventricular white matter at the level of the hippocampus. Additionally, there was marked granulomatous inflammation with severe lymphocytic perivascular cuffing in the right striatal body and caudate nucleus (Figure 2). Moreover, mild lymphoplasmacytic meningitis of the cerebral hemispheres as well as a mild lymphoplasmacytic inflammation of the right optic nerve were detected. No infectious agents were observed using immunohistochemistry (IHC) against rabies virus, canine distemper virus, *Neospora caninum*, and *Toxoplasma gondii*.

### Case 2

A 1-year-old male intact Maltese was presented due to 3-day history of vestibular ataxia. The blood examination of the referring veterinarian was unremarkable. The dog was mildly apathic, but otherwise the general examination was unremarkable. In the neurological examination, mild head tilt and head turn to the left were present. The dog was non-ambulatory and ataxic. Increased extensor-muscle tone was evident in both right limbs. Proprioception (paw placement and hopping) was reduced on both left limbs. The menace response was mildly and inconsistently reduced in both eyes, and occasionally the dog displayed a head tremor. No other cranial nerve deficits were visible at the time of examination. Multifocal intracranial lesions pronounced in the left brain stem and cerebellum were suspected.

On MRI of the brain in general anesthesia, an intraaxial lesion was evident in the white matter of the cerebellum, extending bilaterally into the brain stem over the cerebellar peduncles. In the brain stem, the left side was more affected and involved the nerve roots of the facial, vestibulocochlear, and trigeminal nerves. The lesions were mildly heterogeneous, moderately hyperintense in T2w, and mildly hypointense in T1w. They were ill demarcated, causing a mild mass effect and increased volume of the brain stem. Marked contrast enhancement was evident in the brain stem meninges and ependymal layer of the fourth ventricle, and there was mild to moderate meningeal enhancement in the left temporal lobe. Additionally, there was mild secondary enlargement of the cervical central canal.

Suboccipital CSF contained increased protein content of 135 mg/dl (reference range <25 mg/dl) and a mononuclear pleocytosis (221 cells/μl, reference range <5 cells/μl; 69% monocytes, 30% lymphocytes, and 1% neutrophilic granulocytes) was evident. *Toxoplasma gondii* antibody titer of immunoglobulin (Ig)M and IgG and antibody titer for *Neospora canis* in serum were below 1:32 (immunofluorescence antibody test (IFAT); Laboklin, Bad Kissingen, Germany).

MUO was suspected. Clindamycin 12 mg/kg body weight and prednisolone 0.5 mg/kg body weight *per os* twice daily were given until negative infectious agent titers were confirmed, then clindamycin was discontinued and prednisolone increased to 1 mg/kg bodyweight twice daily.

Initially, clinical signs improved mildly but relapsed and deteriorated after 5 days. Therefore, owners elected euthanasia 2 weeks after the onset of clinical signs.

**Figure 3:**
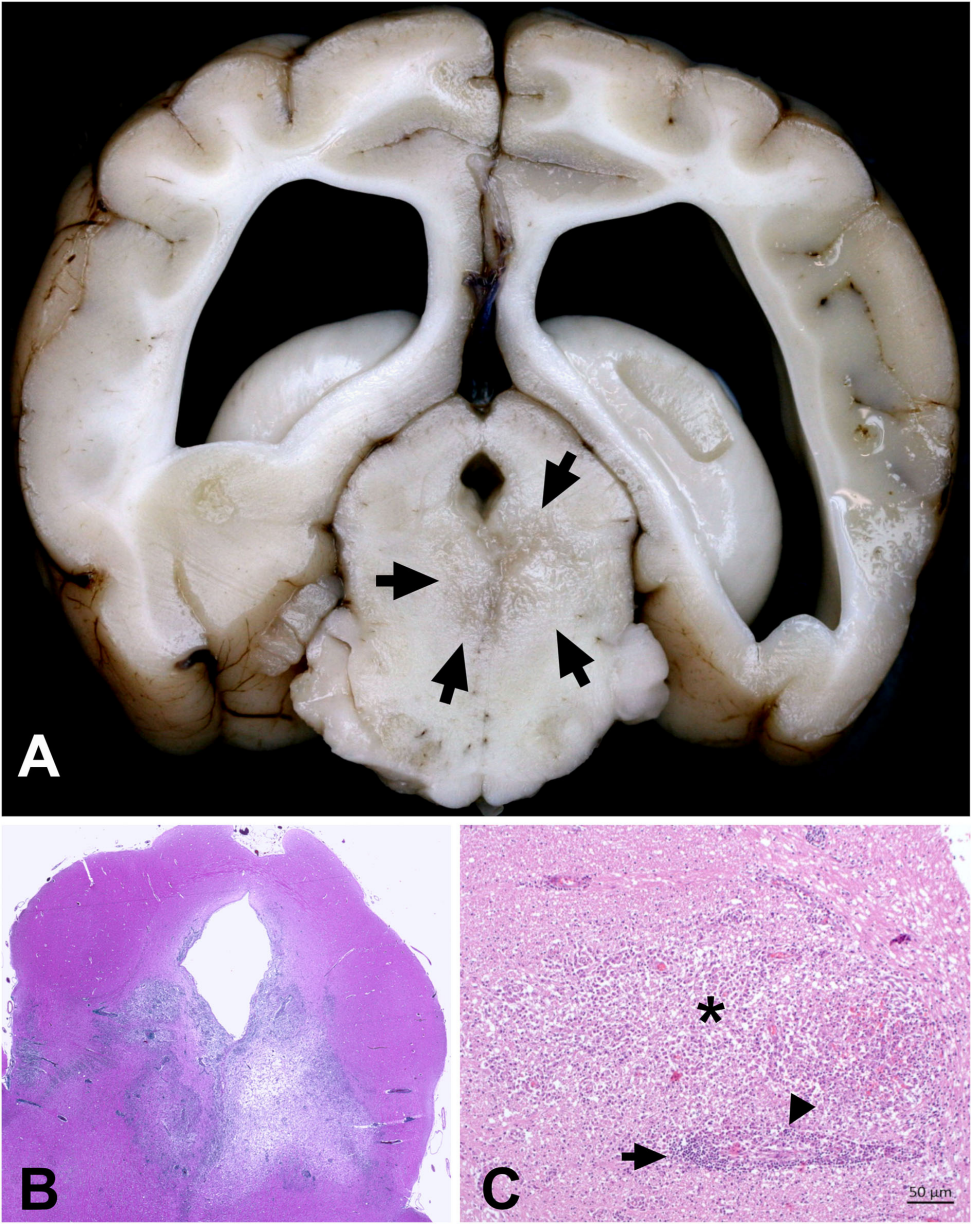
Combined necrotizing and granulomatous meningoencephalitis in the brain of a Maltese (case 2). **(A)** Focal-extensive malacia in the midbrain (arrows). **(B)** Subgross picture of a H&E stained section of the midbrain shown in A. Focal-extensive pallor indicates necrosis and edema, which is associated with a prominent inflammatory process (dark blue color due to nuclei of infiltrating inflammatory cells). **(C)** Area of granulomatous inflammation with lymphocytes (arrows) and activated macrophages (arrowheads) around a vessel. The activated macrophages markedly extend into the neuroparenchyma (asterisk).

On pathological examination, a marked focal-extensive malacia was evident in the area of the cerebellar lingula, brain stem, and midbrain ventral to the aqueduct. On histopathology, extensive necrosis and edema were associated with marked nodular and perivascular infiltration with epithelioid macrophages and lymphocytes in the midbrain, cerebellum, and brain stem (Figure 3). The mesencephalic meninges adjacent to the lesion were also infiltrated by lymphocytes and macrophages. Periodic acid–Schiff (PAS) reaction, Grocott, Gram, and Ziehl-Neelsen staining as well as IHC for *Toxoplasma gondii* and canine distemper virus did not detect any infectious agent.

### Case 3

A 7.5-year-old female Yorkshire Terrier was presented due to status epilepticus. She gave birth to five puppies 4 weeks prior to the presentation, which all died within 3 days *post partum*. She was progressively anorectic for 2 weeks and obtunded. The dog experienced several generalized tonic-clonic cluster seizures for 2 days.

The dog was presented with a generalized tonic-clonic seizure, not responding to external stimuli. The body temperature was normal. Her mucous membranes were pale; general examination was otherwise unremarkable. In the blood examination, mildly increased white blood cell count (18.7 × 103/μl, reference range 4.7–11.3 × 103/μl) with mild lymphopenia was evident with decreased urea 1.6 mmol/L (reference range 3.8–9.4 mmol/L), creatinine 37 μmol/L (reference range 50–119 μmol/L), without any further pathological changes.

On computed tomographic examination of the brain, multifocal, hypoattenuating, and intraaxial lesions were evident in the right piriform and frontal lobes, with moderate mass effect causing a midline shift to the left. Mild heterogeneous contrast enhancement was evident in the lesions. Additionally, there was asymmetrical moderate dilatation of both lateral ventricles.

In suboccipital CSF samples, lymphocytic pleocytosis (88% lymphocytes, 9% monocytes, and 3% neutrophils) was evident. Due to the small sample size, no further examinations could be performed, and the exact cell count was not evaluated.

Seizures could only be poorly controlled with midazolam continuous rate infusion, propofol and phenobarbital bolus injections, and prednisolone therapy, hence her owners elected for euthanasia.

**Figure 4:**
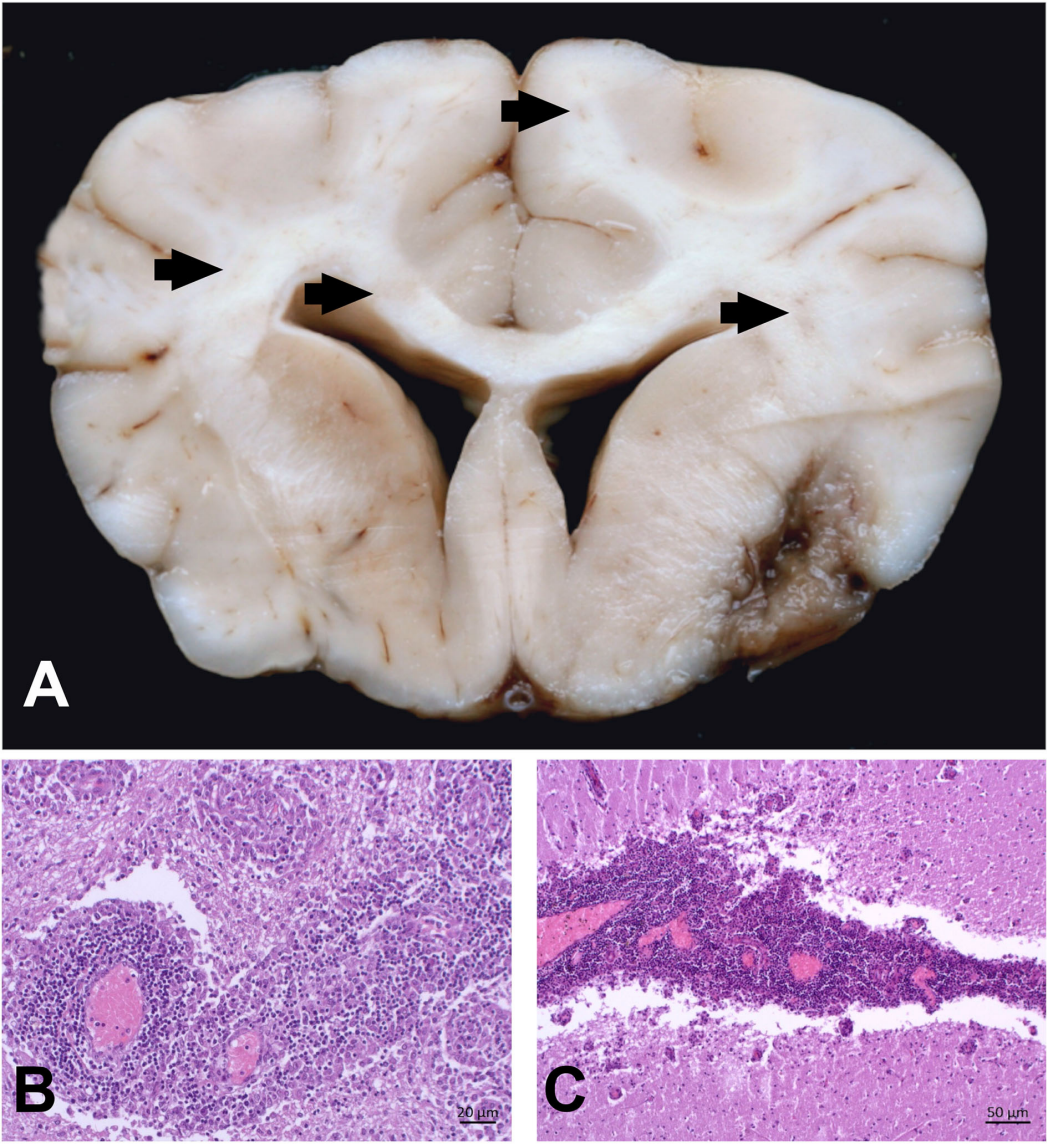
Combined necrotizing and granulomatous meningoencephalitis in the brain of a Yorkshire Terrier (case 3). **(A)** Severe, focal-extensive and delineated malacia in the right rhinal cortex and extending to the surface. This lesion mimicks NME. Additionally, there are multifocal areas of beige discoloration in the corona radiata (arrows). **(B**) Severe and multifocal-coalescing perivascular cuffs with lymphocytes and large activated macrophages. Macrophages tend to compartmentalize within these cuffs. **(C)** The same dog is affected by a marked cerebellar meningitis that extends into the molecular layer.

On pathological examination, multifocal malacic changes were evident in the right piriform lobe, extending to the basal nuclei and meninges, and additionally in the brain stem. On histology, severe inflammation was scattered over the entire brain with multifocal marked distinct perivascular cuffs containing many lymphocytes and macrophages, frequent groups of large epithelioid macrophages, and few plasma cells. Lesions were most evident in the gray and white matter of the frontal lobes and basal nuclei, but they also affected the brain stem, midbrain, thalamus, and hippocampus, and extended into the subarachnoid space (Figure 4). Frontal lobes and basal nuclei were affected by marked multifocal areas of necrosis infiltrated with numerous gitter cells. These lesions were surrounded by numerous gemistocytic astrocytes. The PAS reaction and Grocott staining did not reveal any infectious pathogens.

### Case 4

The formalin-fixed brain of a 7-year-old Chihuahua was sent to the Institute of Pathology in Bern. Before death, the dog had a 4-day history of progressive pacing and circling to the right. No further information was given.

On neuropathology, focal extensive white matter necrosis was evident in the occipital lobes of both hemispheres. Ventral and left of the midline in the midbrain and pons, there was a focal, circular, white-beige, and space-occupying lesion. On histopathology, the space-occupying lesion resembled a granuloma consisting of a central cavitary necrosis, which was intersected with astrocytes and hyperplastic capillaries, surrounded by a thick band of numerous epithelioid macrophages, lymphocytes, and astrocytes. In the cerebral hemispheres, the inflammatory process was more extensive and mainly focused on the white matter. In some areas, the lesions consisted of chronic cavity-like necroses interspersed with gliotic tissue, while in other areas, lesions consisted of active inflammation with perivascular cuffs of numerous epithelioid macrophages, lymphocytes, and fewer plasma cells, intermixed with prominent gemistocytic astrocytes. The inflammatory infiltrates extended multifocally to the gray matter and occasionally to the meninges. The PAS reaction and Ziehl-Neelsen staining did not detect any infectious pathogens.

### Summary

This multicentric case series describes the clinical and pathological findings of two Yorkshire Terriers, one Maltese, and one Chihuahua, with meningoencephalitis of unknown origin. Microscopically, in all dogs, areas of marked necrosis were evident in the cerebral hemispheres (*n* = 3/4; mainly cortical white matter *n* = 2/4, gray and white matter of the frontal lobe and basal nuclei *n* = 1/4), cerebellar white matter (*n* = 1/4), or brain stem (*n* = 1/4) with mainly lymphocytic perivascular infiltrates. Gemistocytes and gitter cells were found in two and three dogs, respectively. At the same time, all four dogs also had focal or multifocal high-grade angiocentric granulomatous inflammatory lesions in the cerebrum (*n* = 2/4; thalamus *n* = 1/2, corpus striatum including basal nuclei *n* = 2/2, cortex *n* = 1/2), and rhombencephalon (*n* = 4/4; brain stem *n* = 3/4, midbrain 2/4, cerebellum *n* =1 /4). Meningitis was found in all the dogs. Infectious agents were excluded using H&E, special staining techniques or IHC.

## Discussion

This case series describes four dogs with histopathological signs of NLE/NME and concomitant GME.

NME, NLE, and GME are often summarized under the umbrella term “MUO” where an unknown multifactorial etiopathogenesis is suspected (1, 2). The different MUO subtypes cannot be reliably differentiated in a clinical setting without histopathological confirmation (2, 11), but in all of them, clinical sings respond to a certain degree to immune-suppressive therapy (2, 10, 11).

Typical histopathological features of GME allow relatively clear differentiation from NME and NLE (13). In GME, angiocentric or nodular granulomatous lesions in the white matter of the cerebrum, cerebellum, medulla oblongata, and spinal cord contain mainly macrophages, epithelioid cells, lymphocytes, plasma cells, and neutrophils (13). In NLE, multifocal lymphoplasmacellular encephalitis and necrotic foci are typical in the cerebral white matter and sometimes in the brain stem (13). For NME, a predominant involvement of the cerebral gray matter and meninges is typical (13). Due to the frequently seen overlap of histopathological and clinical features, as well as MRI findings, NME and NLE are often taken together as NE, and the authors of several reviews have speculated that NME and NLE are rather different manifestations of the same disease entity with breed specific characteristics (1, 14). This study suggests that there might be additionally significant overlaps between GME and NE based on the presented histopathological findings in 4 dogs with concomitant features of GME and NLE/NME. It is already reported that in the absence of necrosis in very acute NME, NME, GME, and NLE might be less easy to distinguish due to overlap in infiltrating inflammatory cell population (13). On the contrary, mild necrotic foci are reported in GME but were not as extensive as in NME or NLE (23).

All dogs were presented with clinical signs of progressive intracranial disease. Signalment, clinical signs, diagnostic imaging, and CSF findings were most consistent with NLE in case 1 and NME in case 2 (14). Although Yorkshire Terriers are predominantly affected by NLE (14), MRI findings of case 3 included an intraaxial lesion with increased contrast enhancement and mass effect, which is more likely to occur in GME than in NLE (14, 24, 25). Due to the incomplete availability of clinical data in case 4, the clinical diagnosis was uncertain. Advanced diagnostic imaging might be rather unspecific for the diagnosis of inflammatory brain lesions (26), and biopsy might be preferred to diagnose MUO subtypes (1, 9).

On histopathology, necrosis and lymphocytic perivascular inflammation in subcortical white matter, in the cerebral cortex, deep gray matter of the telencephalon, or in the brain stem and cerebellum were indicative of NE. Additionally, in all dogs, concomitant granulomatous meningoencephalitis was evident with epithelioid macrophages mostly in the brain stem and also in the telencephalon or spinal cord. In none of the dogs, infectious agents were found with basic histopathological examinations.

Attempts by several working groups to identify an infectious agent in any subtype of MUO failed (4–9, 27). As in this case series, no further in-depth search for infectious agents was performed, e.g., next generation sequencing (4), it cannot be completely ruled out that these dogs might have suffered from a so far unidentified infection, which causes this specific mixed pattern of granulomatous and necrotizing meningoencephalitis. However, immunohistochemistry and special staining for known infectious agents were negative, supporting the classification of MUO.

It is also unknown why some dogs develop GME instead of NME and NLE or vice versa (1, 13). A certain trigger (infectious or environmental) might lead to the development of MUO, and the clinical and histopathological phenotype depends on individual genetic susceptibility. Moon (28) induced experimental autoimmune encephalitis (EAE) in dogs by injecting whole cerebral homogenate subcutaneously together with an inflammatory stimulation agent. While 7 out of 12 dogs developed necrotizing encephalitis, others did not develop any obvious clinical signs (28). The design of the study does not allow any further conclusion on the exact pathogenesis of the encephalitis as the study population was too heterogeneous regarding breed, gender, and even injected brain homogenate derived from different canine donors with various underlying brain disease. Nevertheless, not all dogs, which received brain homogenate from the same donor, did develop EAE (28) pointing to individual predisposing factors.

Additionally, breed-specific disease patterns of MUO are reported (1, 14). The dogs with mixed inflammation patterns of NME/NLE and GME described here were all toy breed dogs, mainly with the necrotizing variants of MUO (14): Maltese dogs and Chihuahuas seem to develop preferably NME (3, 17), while Yorkshire Terriers and French Bulldogs seem to be prone to develop NLE (15, 16, 29). In Maltese dogs and Chihuahua, a genetic defect in dog leukocyte antigen (DLA) class II is suspected to predispose to NME (3). It seems that, among other factors, genetic susceptibility might determine the subtype of MUO. Unfortunately, no statement can be made about the underlying DLA-II genotype of the dogs with mixed GME and NME/NLE in this case series, as no genetic examination was performed.

On the contrary, it seems that different triggers can cause different inflammatory patterns in EAE (30). In the study of Moon, all dogs with EAE, very similar histopathological signs of NLE-like or NME-like lesions were evident after injection of the similar trigger (homogenized cerebral tissue) despite the heterogeneous genetic background in the study population (28). In EAE in rats, injection of homogenized cerebral tissue induced lesions similar to NME in the forebrain, while injection of homogenized cerebellar tissue induced completely different lesions with demyelination and inflammation in the rhombencephalon and spinal cord (30). It seems that both the individual susceptibility and the underlying trigger might have an influence on the expression of a specific EAE subtype (28, 30). The same might also be true for naturally occurring MUO in dogs, and the pattern of concomitant GME and NME/NLE described here might be the result of a particular combination of individual susceptibility and triggers, which differs from classical GME or NME/NLE.

The described pattern of neuroinflammation with concurrent signs of NME/NLE and GME might be either a so far undescribed new subtype of MUO or an atypical variant of either NME, NLE, or GME. Furthermore, it could be hypothesized that NME/NLE and GME are the same disease entity with an individual histopathological expression. Another theory could imply that the presented dogs might have suffered from two different diseases—GME and NE—at the same time, coincidentally.

Although all of these theories are speculative in nature and it cannot be told, what caused this atypical inflammation pattern in the presented dogs, this case series is an additional hint that the subtypes of MUO might not be as distinct as it is generally portrayed, which was already stated by other authors before (1, 14).

Additionally, for *ante mortem* diagnosis of MUO, it is very important to keep in mind that *in vivo* diagnosis of NME/NLE or GME based on biopsy sampling could be misleading: Biopsies only give a focal impression and might not necessarily be representative of the entire inflammatory process in the whole brain. Depending on the side of tissue sampling, a biopsy could lead to an incomplete or even false impression of the overall nature of the inflammation, and either a necrotic or a granulomatous component of the encephalitis could be overlooked in cases of concomitant GME and NME/NLE. The consequence could be an incomplete or false diagnosis, which might have an influence on the results and interpretation of future studies or on the development of clinical therapy strategies.

## Conclusion

Although rarely reported, concomitant signs of GME and NME/NLE can be presented in the diagnostic imaging and histopathological examinations of small toy breed dogs, which should be considered for future studies. Especially in a clinical setting without extensive histopathological examination, the diagnosis MUO should be preferred over the more specific diagnoses of GME, NME, or NLE.

## Data availability statement

The raw data supporting the conclusions of this article will be made available by the authors, without undue reservation.

## Author contributions

JN contributed to conception and design of the study, collected and organized the data, and drafted and finalized the report. AO performed and interpreted results of necropsy, histopathology, and drafted and finalized the report. MS performed neurological examination, interpreted diagnostic imaging findings, and drafted and finalized the report. IS performed and interpreted results of necropsy and histopathology, and drafted and finalized the report. IG performed and interpreted results of necropsy, histopathology, and finalized the report. SB performed neurological examination, interpreted diagnostic imaging findings, and finalized the report. FS performed neurological examination, interpreted diagnostic imaging findings, and finalized the report. MJS performed neurological examination, interpreted diagnostic imaging findings, and finalized the report. AT supervised neurological examination and interpretation of diagnostic imaging findings, contributed to conception and design of the study, and finalized the report. All authors agree to the authorship and the publication of the report. All authors contributed to the article and approved the submitted version.

## Funding

This Open Access study was funded by the Deutsche Forschungsgemeinschaft (DFG, German Research Foundation)−491094227 “Open Access Publication Funding” and the University of Veterinary Medicine Hannover, Foundation.

## Conflict of interest

The authors declare that the research was conducted in the absence of any commercial or financial relationships that could be construed as a potential conflict of interest.

## Publisher's note

All claims expressed in this article are solely those of the authors and do not necessarily represent those of their affiliated organizations, or those of the publisher, the editors and the reviewers. Any product that may be evaluated in this article, or claim that may be made by its manufacturer, is not guaranteed or endorsed by the publisher.

## Abbreviations

CBC: complete blood count
CSF: cerebrospinal fluid
CT: computer tomography
CNS: central nervous system
EAE: experimental autoimmune encephalitis
ECVN: European College of Veterinary Neurology
GME: Granulomatous meningoencephalitis
IFAT: immunofluorescence antibody test
Ig: immunoglobulin
IHC: immunohistochemistry
MRI: magnetic resonance imaging
NE: necrotizing encephalitis
NLE: necrotizing leukoencephalitis
NME: necrotizing meningoencephalitis
PAS: periodic acid–Schiff.
